# Therapeutic Potential of Tea Tree Oil for Scabies

**DOI:** 10.4269/ajtmh.14-0515

**Published:** 2016-02-03

**Authors:** Jackson Thomas, Christine F. Carson, Greg M. Peterson, Shelley F. Walton, Kate A. Hammer, Mark Naunton, Rachel C. Davey, Tim Spelman, Pascale Dettwiller, Greg Kyle, Gabrielle M. Cooper, Kavya E. Baby

**Affiliations:** University of Canberra, Faculty of Health, Bruce, Canberra, Australia; Faculty of Health, University of Tasmania, Hobart, Tasmania, Australia, Faculty of Science, Health, Education and Engineering, University of the Sunshine Coast, Queensland, Australia; School of Medicine and Pharmacology, The University of Western Australia and Translational Renal Research Group, Harry Perkins Institute of Medical Research, Nedlands, Western Australia; Burnet Institute, Melbourne, Victoria, Australia; School of Medicine, Flinders University, Katherine, Northern Territory, Australia; Private Practice, Charnwood, Canberra, Australia

## Abstract

Globally, scabies affects more than 130 million people at any time. In the developed world, outbreaks in health institutions and vulnerable communities result in a significant economic burden. A review of the literature demonstrates the emergence of resistance toward classical scabicidal treatments and the lack of effectiveness of currently available scabicides in reducing the inflammatory skin reactions and pyodermal progression that occurs in predisposed patient cohorts. Tea tree oil (TTO) has demonstrated promising acaricidal effects against scabies mites in vitro and has also been successfully used as an adjuvant topical medication for the treatment of crusted scabies, including cases that did not respond to standard treatments. Emerging acaricide resistance threatens the future usefulness of currently used gold standard treatments (oral ivermectin and topical permethrin) for scabies. The imminent development of new chemical entities is doubtful. The cumulative acaricidal, antibacterial, antipruritic, anti-inflammatory, and wound healing effects of TTO may have the potential to successfully reduce the burden of scabies infection and the associated bacterial complications. This review summarizes current knowledge on the use of TTO for the treatment of scabies. On the strength of existing data for TTO, larger scale, randomized controlled clinical trials are warranted.

## Scabies Infection

### Epidemiology.

Scabies is a contagious, parasitic dermatosis (skin disease) caused by the acarine itch mite *Sarcoptes scabiei* var. *hominis*, affecting 300 million individuals worldwide each year, including all age groups and social classes.^1^–^3^

A World Health Organization (WHO) review estimated a global prevalence of 0.2–24%.^4^ However, the condition is more prevalent in tropical regions, particularly for pediatric scabies. In Australia, scabies is a major public health problem in Indigenous communities, with a prevalence of 25% in adults and about 30–65% in children.^5^,^6^ It is usually contracted by close, prolonged personal contact with an infected person and therefore is very common among family members and often seen in institutional settings.^7^,^8^ It is prevalent among young children and remains frequent in older children and young adults, possibly due to the absence of immunity and increased exposure and cross infection between children.^9^,^10^

### Disease morbidity.

**Figure 1.**
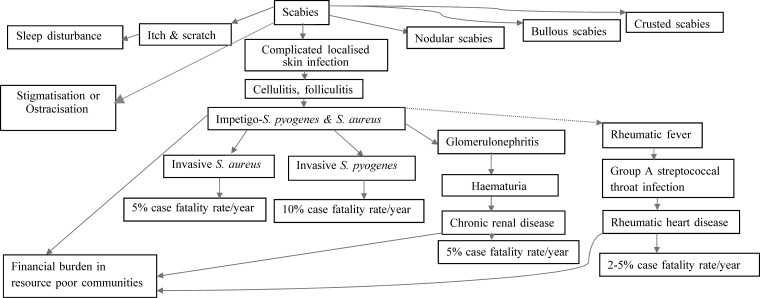
Complications of scabies infection, modified from Engelman and others.^3^

*Sarcoptes scabiei* releases antigens that diffuse into the outer skin layer resulting in local inflammatory and immune reactions, leading to severe pruritus and skin abrasion.^11^,^12^ Breaks in the epidermis serve as an entry point for pathogenic bacteria (usually streptococci or staphylococci), which complement inhibitors from scabies mites and promote bacterial growth.^13^ The scabies mites secrete a number of endogenous molecules that inhibit the host immune system.^13^,^14^ This process is believed to protect the invaded mites from host defense mechanism. Molecular studies have also revealed that scabies mites produce a variety of endogenous complement inhibitors (e.g., scabies mite–inactivated protease paralogues Il and Dl and scabies mite serpins such as SMSB3 and SMSB4), which interfere with different stages of host defense compliment cascades and promote the formation of bacterial pathogens (e.g., *Staphylococcus aureus* and *Streptococcus pyogenes*) in the patient's body favoring the establishment of secondary bacterial coinfections.^14^–^16^ Superinfected lesions may develop into cellulitis or impetigo and may contribute to abscess formation. These sequelae predispose the infected individuals to sepsis and other nonsuppurative invasive infections, as explained in Figure 1

.^17^ A more severe or “crusted” form of infestation is associated with extreme incapacity and with disorders of the immune system, for example, human immunodeficiency virus infection.

In Australia, the Indigenous population has been found to suffer from streptococcal septicemia (with infectious diseases driven by impetigo and associated scabies being the major cause in rural and remote areas) at a five times greater rate than the general population.^18^ This contributes to an estimated life expectancy gap of 13 years (2012 data) between Indigenous and non-Indigenous Australians.^19^ Furthermore, a clear link between scabies and bacterial pyoderma has been identified as the causative factor for rheumatic fever and heart disease, skin sepsis, and renal disease in Aboriginal and Torres Strait Islander communities in Australia.^20^

### Economic burden of disease.

A 2004 U.S. study estimated the annual economic burden for scabies management at US$10.4 million.^21^ In Australia, the estimated annual (2013 data) cost associated with the management of pediatric scabies and pyoderma per patient was AU$10,000. This is the minimum cost associated with hospitalizations as no further data or comprehensive estimates of the annual economic burden are available.^6^,^22^,^23^

### Current treatments.

Topical treatments for scabies include sulfur, benzyl benzoate, allethrin, thiabendazole, crotamiton, monosulfiram, malathion, lindane, and permethrin. Oral treatments are limited to ivermectin. All scabies treatments are potentially hazardous and associated with moderate to severe side effects (Table 1).^2^,^8^,^41^,^45^–^47^ The most frequent complication of topical scabicides is persisting post-scabies eczema (generalized eczematous dermatitis) from the various formulations.^12^ Further, it may be difficult to treat patients with secondary eczematization, erosions, or ulcers using topical scabicidal agents, as they can cause serious cutaneous and systemic side-effects, resulting in poor treatment uptake.^48^

Ivermectin (the sole oral treatment) is delivered to the infective organisms via ingested intraepidermal fluids. Hence, young children, the elderly (particularly women), individuals with asteatotic skin (e.g., taking estrogens or retinoids), and diabetic individuals are at increased risk for treatment failure due to minimum sebum production.^49^ No currently available acaricides for scabies possess ovicidal activity, so re-treatment is sometimes needed, to kill newly hatched mites. This problem is further exacerbated by increasing acaricide resistance leading to treatment failures.^50^

### Acaricide resistance.

In vitro and in vivo studies completed in Australia and elsewhere have raised concerns about increasing resistance to ivermectin (Australian authors reported strains that were totally resistant to ivermectin) and permethrin.^1^,^31^,^51^–^55^ A 1994 in vitro study reported 100% mortality of scabies mites after 1 hour exposure to 5% permethrin; however, a study conducted 6 years later showed > 3-fold increase in tolerance to permethrin.^1^,^7^,^50^,^56^ More recent studies have confirmed that permethrin is now the slowest acting acaricide in vitro in this region (Northern Territory, Australia).^50^ A 2013 review article cited seven incidents of ivermectin resistance leading to treatment failures in northern Australia.^7^ Clinical and in vitro ivermectin resistance has also been documented in crusted scabies patients.^7^ In vitro sensitivity data from the past 10 years indicate that median survival times for ivermectin have doubled since its introduction.^56^ A 2009 clinical trial in Senegal reported poor therapeutic response to ivermectin for the management of scabies in children. There was a cure rate of 24.6% with a single dose of ivermectin 150–200 μg/kg, although the study has been criticized for the variability of dose administered. The authors have also now indicated the possibility of ivermectin resistance in the treated patient cohort resulting from the previous use of ivermectin in the mass treatment program for onchocerciasis, a parasitic disease also known as river blindness caused by filarial worm *Onchocerca volvulus*.^57^ Further, when ivermectin is used alone, it is not effective against crusted scabies, requiring coadministration of topical agents such as scabicidal preparations or keratolytics. In addition, resistance to other acaricides, such as lindane and crotamiton, has also been reported worldwide.^1^,^3^,^7^,^46^,^56^,^58^

An ideal acaricide would possess ovicidal, antibacterial, anti-inflammatory, and/or antipruritic properties, and would be effective in preventing treatment relapse (resulting from newly hatched mites), inflammatory skin reactions (from mite antigens), and pyodermal progression. It would have a low incidence of resistance and would not contribute to the development of resistance to other agents. The long-term usefulness of ivermectin and permethrin for the treatment of scabies is uncertain because they fail to meet these requirements. It is doubtful that new chemical entities will be developed in the near future. Though there may be potential for immunological control, the development of a vaccine may be decades away.^7^ Veterinary vaccines are available (e.g., TickGARD, GAVAC) for the management of ectoparasitic conditions such as cattle tick (*Boophilus microplus*).^59^ Development of a scabies vaccine seemed feasible, since animals that recover from the infection possess protective immunity against mite reinfestation.^60^–^62^ Vaccination using dust mite extracts provided protection for immunized animals from mite challenge^63^; however, several obstacles have hindered development of a scabies vaccine.^63^ Further studies are required to identify the protective antigens and/or antibodies, and deliver a detailed understanding of how the body's immune system controls scabies, including increased understanding of immune pathogenesis of crusted scabies.^63^ Preexisting immune responses to selected antigens in endemic areas (e.g., indigenous communities in Australia) also need careful consideration.^63^,^64^ Compliance to vaccination could be a potential limiting factor in community settings; latest advancements in the field such as needle-free skin vaccination could be an attractive option to administer vaccine in mass immunization programs to eradicate this highly debilitating infection.^63^,^65^

Another important consideration is that patients with crusted scabies are often identified as core transmitters of scabies to others in the community, and therefore the spread of acaricide-resistant mites may jeopardize the future of current treatment options.^58^,^66^ There is clearly a need for further clinical studies to assess alternative treatments that have shown excellent results in preliminary in vitro studies. Botanicals have been identified for the management of infectious skin conditions and a partial summary of the key candidates is summarized in Table 2.^67^,^68^ This topic has been extensively reviewed and updated in recent publications.^68^,^69^ Of these botanicals, tea tree oil (TTO) is an ideal candidate for research.^1^

## Tea Tree Oil

TTO is documented as having been used in the community (in Australia and internationally) for over 90 years.^70^,^71^ Indigenous populations have been using this plant, *Melaleuca alternifolia*, and derivatives for far longer. TTO has been found to be effective (in vitro) as a bactericide (at 0.002–2%; including against MRSA [methicillin-resistant *S. aureus*]), fungicide (0.004–0.25%), and as an anti-inflammatory agent (≤ 0.125%).^70^,^71^ It has been used in reducing MRSA colonization and in the treatment of a wide range of bacterial, fungal, and viral skin infections. It has also been used as a topical antipruritic agent.^70^–^72^ The therapeutic benefits of TTO-containing formulations for a range of dermatological conditions have been investigated in several randomized controlled trials (RCTs), which have demonstrated safety and efficacy in the general population (noted studies are summarized in Table 3).^70^,^71^ Levels of components in TTO are specified under an International Organization for Standardization standard (ISO 4730), reducing the potential for compositional variation, which is often noted as a problem with botanical medicinal products.^70^,^71^

### Antibacterial activity.

The potent antibacterial activity of TTO has received much attention, highlighting its potential usefulness as a topical antibacterial agent.^70^,^71^ Minimum inhibitory concentrations (MICs) range from approximately 0.06–0.5% for a wide range of gram-positive and gram-negative bacteria, with the exception of *Pseudomonas aeruginosa*, which has MICs in the range of 2–8%.^75^ Of note is that TTO is equally active against antimicrobial resistant and susceptible strains, such as MRSA and methicillin-susceptible *S. aureus*.

### Anti-inflammatory activity.

Terpinen-4-ol, at concentrations equivalent to 0.125%, can inhibit the production of several inflammatory mediators, such as tumor necrosis factor alpha, interleukin-1β, and prostaglandin E_2,_ as well as superoxide production, resulting in diminished inflammatory response.^70^ TTO has been shown to reduce hypersensitivity responses in the skin including responses to insect bites, bee stings, hives, and metal-induced hypersensitivity.^76^ This is chiefly attributed to the ability of TTO to modulate the vasodilation and plasma extravasation associated with histamine-induced inflammation.^77^

Application of the topical scabicide benzyl benzoate is typically associated with a burning sensation, and in children it needs to be diluted to reduce the severe stinging sensation. Dilution compromises its potency and efficacy. The incorporation of TTO (5%) into a commercial product (Ascabiol^®^, 25% benzyl benzoate) has been found to improve the tolerability of the product, chiefly attributed to the anti-inflammatory activity of TTO components.^50^,^78^

### Antipruritic activity.

TTO has also demonstrated benefit in reducing pruritus in human and animal studies.^72^,^79^–^81^ However, large scale RCTs to further explore the antipruritic efficacy of TTO-containing formulations have not been performed. Sufficient anecdotal evidence exists to warrant comparing TTO formulations against active comparators for the therapeutic management of pruritic skin conditions.

## Preliminary Data/Pilot Studies

### Insecticidal, acaricidal, and repellent effects of TTO.

TTO has shown insecticidal, acaricidal, and repellent effects against a range of medical and veterinary pests when compared with commercial preparations both in vitro and in vivo, including whitefly,^82^ head lice (*Pediculus humanus* var. *capitis*),^73^ and sheep lice.^83^

### Acaricidal effects.

In addition to its broader insecticidal activity, TTO has also demonstrated promising potential as an acaricide in numerous in vitro and in vivo exploratory studies. House dust mites (*Dermatophagoides pteronyssinus*) showed 100% mortality in vitro after exposure to a 10% TTO formulation.^84^ Face mites (*Demodex folliculorum*) survived only 3.7 minutes after in vitro treatment with 100% TTO, and 14.8 minutes after 50% TTO compared with no mortality at the maximum observation time of 150 minutes after treatment with 10% povidone iodine or 4% pilocarpine.^85^ In an in vivo trial using a daily eye-lid scrub containing 50% TTO, there was a 78% cure rate (*N* = 7/9) at 4 weeks and no recurrence 1 month later^85^,^86^; Swine mange mite (*S. scabiei* var. *suis*) infestation was resolved in 98.5% of cases 4 weeks after treatment completion in an in vivo field trial using two applications of 1% TTO a week apart.^87^ The in vitro scabicidal activity of TTO against human scabies mites, *S. scabiei*, demonstrated a superior result in comparison with standard treatments (150 minutes with ivermectin 100 μg/g; 120 minutes with permethrin 5%, compared with 60 minutes median survival time with 5% TTO).^1^,^78^ TTO 5% has also been used on an ad hoc basis at the Royal Darwin Hospital (Darwin, Northern Territory, Australia) in combination with benzyl peroxide and oral ivermectin (200 μg/kg) for the management of complicated crusted scabies (two to three times per week for 1–4 weeks, depending on the disease severity)^88^ and in patients who did not initially respond to oral ivermectin therapy.^1^

### Safety, tolerability, and stability.

Topical application of TTO is associated with a low incidence of adverse effects, mostly irritant or allergic reactions to the oil. Irritant reactions can be largely avoided through the use of products containing lower concentrations of oil. Although the threshold for irritant reactions has not been determined, it seems they are rarely associated with TTO concentrations less than 20%. When the oil was formulated in a suitable pharmaceutical base (cream/ointment/gel) containing concentrations of 25% or less and was applied once daily for 21 consecutive days (*N* = 311, adults), it caused no skin irritation.^89^ Allergic reactions may occur even at very low concentrations and can be confirmed using patch tests. The incidence of positive patch tests to TTO in consecutive patch-tested patients attending specialist dermatology or immunology clinics is approximately 0.03%.^90^ Another study, in which 217 patients from a dermatology clinic were patch tested with 10% TTO, found no irritant reactions.^91^

The potential for TTO toxicity in children is yet to be evaluated extensively. A recent RCT investigated the use of TTO (75%) for the management of the viral infection molluscum contagiosum in children (mean age = 6.3 + 5.1 years, 30 days treatment).^92^ TTO was found to be well tolerated in the treatment cohort. Another study showed that the irritation potential of TTO resulted from the oxidation of oil leading to elevated levels of peroxide and 1,2,4- trihydroxy menthane.^93^ 1,2,4-Trihydroxy menthane is a degradation product of TTO and a known skin sensitizer.^94^ Neat TTO is sold in amber glass bottles fitted with child-resistant polypropylene caps and is recommended to be stored at 22°C away from direct heat and light. In typical in-use conditions, TTO will have no appreciable degradation for up to 12 months.^89^,^93^

The chemical composition of TTO has been widely studied and well defined. TTO consists largely of cyclic monoterpenes, of which about 50% are oxygenated and 50% are hydrocarbons.^95^ The international standard ISO 4730 for *Melaleuca*, terpinene-4-ol type (TTO) contains three major components: terpinen-4-ol, γ-terpinene, and α-terpinene comprising about 70% of whole oil, while ρ-cymene, terpinolene, α-terpineol, and α-pinene account for about 15% of the oil.^95^,^96^ Because of high volatility, 90% of the TTO is removed quickly from the skin surface, minimizing the potential for TTO components to travel into the deeper layers of the skin and to be absorbed into the bloodstream.^97^ Under nonoccluded conditions, penetration of TTO components through the skin is limited but terpinen-4-ol and α- terpineol (the chief bioactive constituents^96^,^98^) are able to penetrate the epidermal layer (3.6–8.0% and 3.6–8.4% of the applied amount, respectively, over 25-hour period after application of the pure oil^97^) of the skin sufficiently to provide antimicrobial, anti-inflammatory, and acaricidal effects.^70^,^99^ However, when 20% TTO formulation (in ethanol) was tested, only terpinene-4-ol (< 0.05% of the applied formulation) was able to fully penetrate the epidermis.^97^

#### Resistance to TTO.

Since its introduction in the 1920s, resistance to TTO per se has not yet been reported. It has been postulated that the multiple active components in TTO may reduce the potential for resistance to occur spontaneously, since multiple simultaneous mutations would be required to overcome all the actions of the individual components.^70^ TTO is known to affect multiple properties and functions associated with the cell membrane (similar to membrane-active biocides), meaning numerous targets would have to adapt to overcome the effects of the oil.^70^

## Discussion

Scabies was listed as a neglected tropical disease by the World Health Organization (WHO) in 2013.^100^ Preventing and decreasing morbidity associated with scabies infestation is a national public health priority in some countries. WHO has called for an international alliance for research into the control of scabies. In the developed world, outbreaks in health institutions and vulnerable communities result in a significant economic burden.^17^ In 2010, it was estimated that the direct effects of scabies infestation on the skin alone led to more than 1.5 million years lived with disability, and the indirect effects of complications on renal and cardiovascular function were found to be far greater. The antibacterial properties, together with wound healing effects,^101^,^102^ of TTO could prevent the disease progression to pyoderma, secondary sepsis, and other suppurative and nonsuppurative bacterial complications associated with scabies infestation, especially in children. Furthermore, the anti-inflammatory and antipruritic activity of TTO could reduce the inflammatory immune reactions seen as a response to mite antigens.

Preclinical investigations have demonstrated superior scabicidal properties of TTO when compared with widely used scabicidal agents, such as permethrin 5% cream and ivermectin. Hence, it is reasonable to assume that a TTO-containing formulation (≥ 5%) could be useful for the management of scabies infestations in humans and would be less likely to cause cutaneous and/or systemic side effects. Intuitively, this approach could lead to the development of a topical treatment option for the therapeutic management of ectoparasitic infestations in humans and in animals (“one health approach”). TTO formulated for topical use would have the added advantage of being cost-effective, simple to use, and could be implemented in remote communities as a traditional medicine for the long-term management of scabies and pyoderma in children.

Despite the fact that TTO has a relevant spectrum of activity against scabies' mites, proven antimicrobial and anti-inflammatory activities, putative anti-itch properties, and promising preliminary clinical evidence of safety and effectiveness, it is unlikely to be investigated and developed as a treatment of scabies by the usual commercial mechanisms. This is because the intellectual property associated with these properties is already in the public domain and TTO would not meet the novelty requirement for a patent. Although patents are not the only mechanism that may be used to protect intellectual property, they are a cornerstone of the commercial drug development process.^103^ Without a patent or other similar means to protect the intellectual property associated with developing and using TTO to treat scabies, there is little commercial incentive for anyone to bear the risk and cost associated with product development and safety and efficacy testing.^103^ Consequently, the evaluation of a potentially useful, low-cost, nonproprietary treatment may continue to be overlooked. Ironically, the burden of scabies disease is borne disproportionately by the poor,^104^ a group likely to benefit the most from the availability of nonproprietary treatments such as TTO.

## Figures and Tables

**Table 1:** An overview of classical treatments indicated for the management of scabies in Australia

Drugs	Dosage	Treatment regimen	Contraindication	Disadvantages	Indicative cure rates	Comments
Topical						
Benzyl benzoate	25% solution	One or several consecutive 24-hour applications	Pregnant women and infants	Burning or stinging, pruritus, dermatitis	86% (72/86)^24^; daily application for three consecutive days; cure rate at week 4	In use since 1930s; neurological complications with misuse; withdrawn in the European Union due to neurotoxicity concerns
Permethrin	5% cream	Apply overnight (8–14 hour) then wash off	Infants aged < 2 months	Mild burning, itching stinging, pruritus, erythema, tingling, rash, diarrhea, persistent excoriation, dystonia (rare),^25^ convulsions (rare)	96.3% (106/110)^26^; permethin 2.5%, twice in 1 week; cure rate at week 4	In use since the 1980s; relatively expensive; growing resistance to scabies mites; poor compliance reported in mass community intervention programs
Sulfur	2–10% precipitate in petroleum base	Apply for 24 hours, and then wash and reapply repeat applications for 3 days	–	Noxious, malodorous messy; not given as first-line agents; multiple applications required; can cause skin irritation	96.9% (31/32)^27^; 8–10% three consecutive days, cure rate at week 4	Has been used for centuries; indicated in infants, pregnant and lactating women; inexpensive
Oral						
Ivermectin	200 μg/kg orally repeated after 1–2 weeks	–	Children < 15 kg; children aged < 5 years; pregnant or lactating women	Transient side effects: gastrointestinal disorders; pustular rash, cellulitis; abdominal pain, diarrhea, headache, vomiting, hypotension, toxic epidermal necrosis, mucosal drug eruption, fever, anorexia, lymph node swelling, eosinophilia, pain of joint and muscles, mazzotti reaction^28^	43.1% (28/65)^29^; single dose, 150–200 μg/kg; cure rate at week 4	In use since 1980s (for the mass treatment of onchocerciasis, and filariasis); not approved for the treatment of typical scabies (except in Japan, Brazil, and France); only indicated if symptoms persists 3 weeks after application of benzyl benzoate or permethrin; no ovicidal activity, thus repeat treatment is required; one report of increased deaths^30^ among elderly patients during scabies outbreak in an institutional setting

Adapted from References.^28^–^45^

**Table 2:** A partial summary of various plant-derived treatments used for the management of infectious dermatological conditions

Botanicals	Pharmacological claims
*Achyranthes aspera* (Amaranthaceae)	Traditionally used for the management of scabies
*Allium cepa* (Liliaceae)	Management of fungi-associated skin diseases
*Aloe vera* (Xanthorrhoeaceae)	Antibacterial and antifungal properties
Arborvitae (*Thuja occidentalis*; Cupressaceae)	Treatment of verruca vulgaris
Beard lichen (*Usnea barbata*; Parmeliaceae)	Antibacterial activity against gram-positive bacteria
*Cannabis sativa* (Cannabaceae)	Crushed leaves used for the management of scabies
Celandine (*Chelidonium majus*; Papaveraceae)	Treatment of warts
*Coriandrum sativum* (coriander oil; Apiaceae)	Antibacterial properties; treatment of inflammatory skin conditions with bacterial colonization
*Echinacea purpurea*, *Echinacea angustifolia* (Asteraceae)	Traditional oral remedies for warts
Epigallocatechin gallate (standard green tea extract; Theaceae)	Management of external genital or perianal warts
*Eucalyptus globulus* (Myrtaceae)	Management of facial demodicosis
*Eucalyptus pauciflora* (essential oil of snow gum; Myrtaceae)	Strong antifungal activities against a broad-spectrum funding including dermatophytes
*Euphorbia wallichii*, *Euphorbia hirta*, *Euphorbia tirucalli* (Euphorbiaceae)	Activity against gram-positive bacteria and fungi
*Ficus carica*, *Ficus racemosa*, *Ficus benghalensis* (Moraceae)	Management of warts and scabies
Garlic (*Allium sativum*; Amaryllidaceae)	Key active ingredient (ajoene) possess antifungal properties
Hyperforin (*Hypericum perforatum* [Saint John's wort]); Clusiaceae)	Antibacterial activity against gram-positive bacteria
Japanese herbal medicine (Kampo medicine)	Antibacterial against *Propionibacterium acnes*, *Staphylococcus epidermis*, and *Staphylococcus aureus*
*Lawsonia inermis* (Lythraceae)	Treatment of impetigo
Lemon balm (*Melissa officinalis*; Lamiaceae)	Antiviral properties
*Leptospermum scoparium* (Myrtaceae)	Antibacterial, antifungal, and anti-inflammatory properties
*Mangifera indica* (Anacardiaceae)	Treatment of scabies
*Melaleuca alternifolia* (tea tree oil; Myrtaceae)	Antibacterial, antifungal, and antiparasitic properties
Olibanum (*Boswellia serrata*; Burseraceae)	Antibacterial activity against gram-positive bacteria
*Plumbago zeylanica* (Plumbaginaceae)	Treatment of ringworm
Podophyllotoxin (*Podophyllum peltatum*; Berberidaceae)	Management of condyloma acuminata (anogenital wart)
*Rosmarinus officinalis* (rosemary oil; Labiatae)	Antibacterial activity against gram-positive bacteria
Sage (*Salvia officinalis*; Lamiaceae)	Antibacterial activity against gram-positive bacteria
*Sarcococca* (Caesalpiniaceae)	Treatment of scabies and tinea pedis
Siberian ginseng (*Eleutherococcus senticosus*; Araliaceae)	Traditional oral remedies for warts
*Melaleuca alternifolia* (Myrtaceae)	Activity against bacterial, viral, fungal, and protozoal infections affecting skin
*Thyme vulgaris* (Lamiaceae)	Treatment of bacterial skin infections

Adapted from References.^67^,^68^

**Table 3:** Selected randomized controlled trials of TTO in dermatology

Author, year, origin, design	Population and size	Results
Enshaieh and others, 2007, Iran, RCT	*N* = 60, 15–25 years with mild to moderate facial acne	The treatment group (5%, TTO gel) was 5.8 times more effective than placebo (*P* > 0.05)
Carson and others, 2001, AUS, RCT	*N* = 16, 18–70 years with a self-reported history of recurrent herpes labialis	In the treatment group (6% TTO gel), median time for reepithelialization was 9 days vs. 12.5 days for placebo (*P* > 0.5)
Satchell and others, 2002, AUS, RCT	*N* = 126, 14 years and above with mild to moderate dandruff	5% TTO shampoo showed a 41% improvement in the severity score compared with 11% in the placebo group (*P* < 0.001)
Dryden and others, 2004, AUS, RCT	*N* = 236, adults colonized with MRSA	TTO preparations (10% cream, 5% body wash) were more effective than chlorhexidine or silver sulfadiazine at clearing skin lesions
Tong and others, 2007, AUS, RCT	*N* = 121, adults with clinically diagnosed tinea pedis	Mycological cure rate was 64% in the 50% TTO group compared with 31% in the placebo group
Barker and others, 2010, AU, RCT	*N* = 123, children 4–12 years infested with live head lice	The pediculicide-containing TTO and lavender oil (10% TTO and 1% lavender oil) showed 97.6% effectiveness (louse-free subjects) as opposed to 25.0% by the commercial product containing pyrethrins (1.65 mg/g) and piperonyl butoxide (16.5 mg/g)
Blackwood and others, 2013, Ireland, RCT	*N* = 445, adults admitted to intensive care facilities	TTO (5%) group showed no difference (*P* > 0.5) to standard care (Johnson's Baby Softwash) in preventing MRSA colonization

AUS = Australia; MRSA = methicillin-resistant *Staphylococcus aureus*; TTO = tea tree oil.

Adapted from Carson and others,^70^ Barker and others,^73^ and Blackwood and others.^74^
